# Gonadal white adipose tissue is important for gametogenesis in mice through maintenance of local metabolic and immune niches

**DOI:** 10.1016/j.jbc.2022.101818

**Published:** 2022-03-10

**Authors:** Chao-Fan Yang, Wen-Wen Liu, Hai-Quan Wang, Jia-Le Zhang, Kang Li, Zhen-Yu Diao, Qiu-Ling Yue, Gui-Jun Yan, Chao-Jun Li, Hai-Xiang Sun

**Affiliations:** 1 Ministry of Education Key Laboratory of Model Animal for Disease Study and Center for Reproductive Medicine, Department of Obstetrics and Gynecology, Nanjing Drum Tower Hospital, The Affiliated Hospital of Nanjing University Medical School, Nanjing University, Nanjing, China; 2 State Key Laboratory of Reproductive Medicine and China International Joint Research Center on Environment and Human Health, Center for Global Health, School of Public Health, Nanjing Medical University, Nanjing, China

**Keywords:** gametogenesis, gWAT, lipectomy, immune niche, metabolic niche, BTB, blood–testis barrier, cDNA, complementary DNA, E2, estradiol 2, eWAT, epididymal white adipose tissue, FSH, follicle-stimulating hormone, gWAT, gonadal white adipose tissue, HTF, human tubal fluid, LH, luteinizing hormone, PC, phosphatidylcholine, PE, phosphatidylethanolamine, pWAT, periovarian white adipose tissue, T, testosterone, TNF-α, tumor necrosis factor α, qPCR, quantitative PCR

## Abstract

Gonadal white adipose tissue (gWAT) can regulate gametogenesis *via* modulation of neuroendocrine signaling. However, the effect of gWAT on the local microenvironment of the gonad was largely unknown. Herein, we ruled out that gWAT had a neuroendocrine effect on gonad function through a unilateral lipectomy strategy, in which cutting off epididymal white adipose tissue could reduce seminiferous tubule thickness and decrease sperm counts only in the adjacent testis and epididymis of the affected gonad. Consistent with the results in males, in females, ovary mass was similarly decreased by lipectomy. We determined that the defects in spermatogenesis were mainly caused by augmented apoptosis and decreased proliferation of germ cells. Transcriptome analysis suggested that lipectomy could disrupt immune privilege and activate immune responses in both the testis and ovary on the side of the lipectomy. In addition, lipidomics analysis in the testis showed that the levels of lipid metabolites such as free carnitine were elevated, whereas the levels of glycerophospholipids such as phosphatidylcholines and phosphatidylethanolamines were decreased, which indicated that the metabolic niche was also altered. Finally, we show that supplementation of phosphatidylcholine and phosphatidylethanolamine could partially rescue the observed phenotype. Collectively, our findings suggest that gWAT is important for gonad function by not only affecting whole-body homeostasis but also *via* maintaining local metabolic and immune niches.

As endocrine function has been elucidated, white adipose tissue has been shown to be closely related to infertility in the reproductive system (1, 2). An adequate supply of energy ensures the reproductive process is carried out properly (3, 4). A decrease in adipose tissue in different diseases, such as anorexia nervosa (5), lipodystrophy (6), and hypothalamic amenorrhea (7), is related with a series of pathological changes in the reproductive system, including nonobstructive azoospermia and infertility in males (8) and menstrual irregularities, polycystic ovary syndrome, and enhanced rates of miscarriage in females (9, 10).

As an important organ closely connected to the male reproductive system, epididymal white adipose tissue (eWAT) appears to have a special role in support of reproductive status. Previous research showed that removal of eWAT associated with the testis leads to inhibition of spermatogenesis in frogs (11) and rats (12). Similar to the phenotype found in Syrian hamsters, surgical removal of eWAT caused decreased testicle weight and sperm counts but did not affect the mating behavior of male hamsters (13). In female mice, proper storage of fat is also important for oogenesis, and removal of periovarian white adipose tissue (pWAT) adjacent to the ovaries led to a decrease in serum follicle-stimulating hormone (FSH) and estradiol 2 (E2) levels, an increase in luteinizing hormone (LH) levels, and a disturbance in the ovulatory cycle of the mice (14). In brief, these data indicated that lipid depots near the testis or ovary are essential for normal reproductive system function.

Currently, most research suggests that the connection between gonadal white adipose tissue (gWAT) and the reproductive system is mainly regulated by systemic neuroendocrine alterations. Adipose tissue can secrete various adipokines, including adiponectin, lipocalin-2, tumor necrosis factor α (TNF-α), leptin, and interleukin 6, which can regulate glucose/lipid metabolism, inflammation, cell growth, and apoptosis (15, 16). A lack of these factors can disturb the hypothalamic–pituitary–gonadal axis, leading to abnormal spermatogenesis (17) in males or unsuccessful hypothalamic anovulation (18) in females.

In addition to influencing systemic neuroendocrine activity, we hypothesize that gWAT can also affect the local microenvironment in the testis or ovary. Normally, adipose reduction can be directly achieved *via* lipectomy or liposuction (11, 19). We ruled out the influence of the systemic circulatory system through unilateral resection; that is, one side of gWAT was removed, and the same mouse was subjected to sham surgery on the contralateral adipose tissue. We aimed to examine the function of gWAT in the local microenvironment of the reproductive system and the process of gametogenesis.

## Results

### Unilateral lipectomy does not alter body homeostasis but affects adjacent gonads

**Figure 1:**
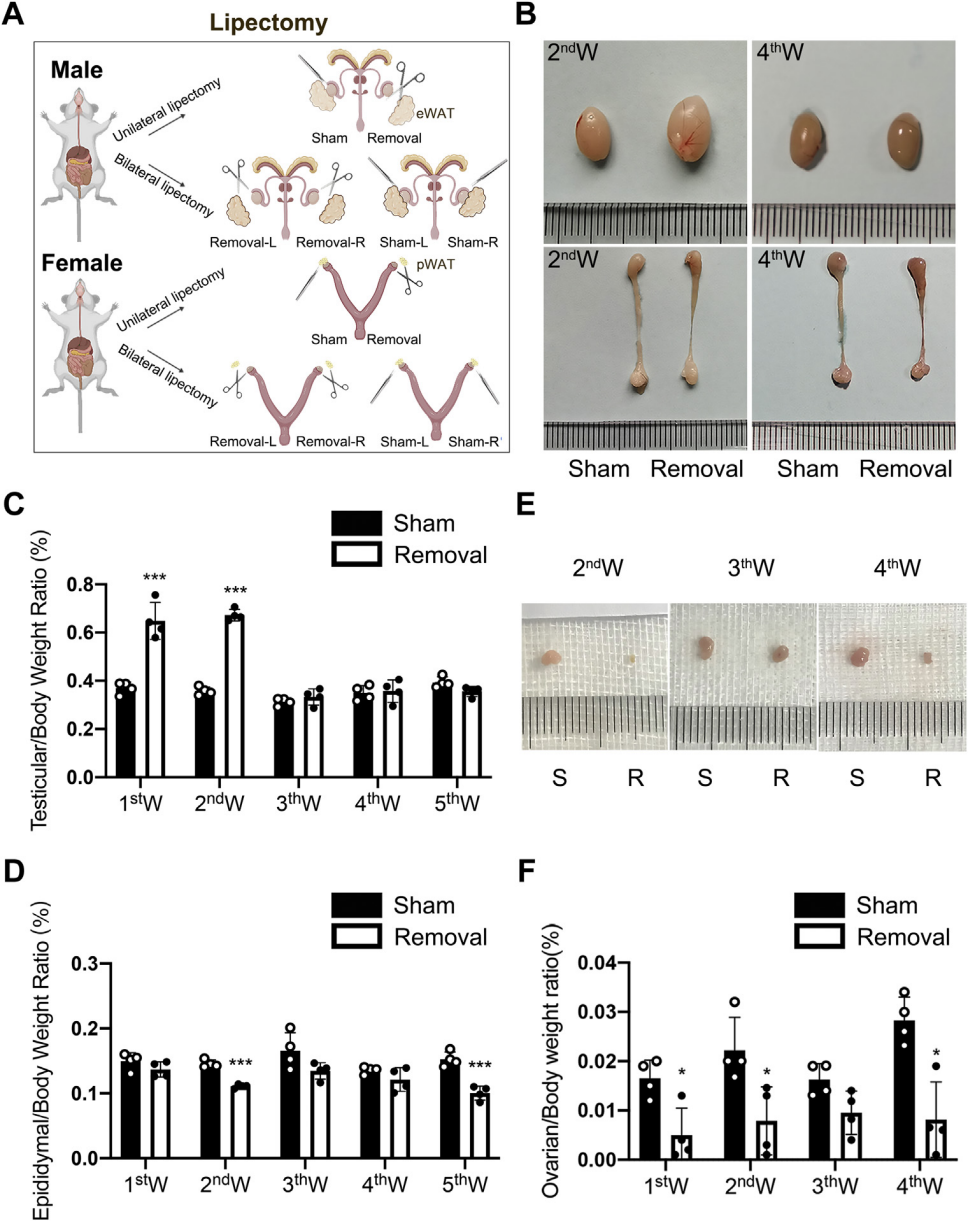
**Lipectomy leads to the reduction of ovarian mass and epididymal mass, whereas testis mass first increases and then decreases.***A*, unilateral lipectomy and bilateral lipectomy of gWAT. Unilateral lipectomy refers to the sham surgery on the left, whereas it refers to the removal surgery on the right in the same mice. Bilateral lipectomy including bilateral removal surgery and bilateral sham surgery. *B*, representative images of testes and epididymis in 8-week-old mice at the second and fourth weeks after unilateral lipectomy. *C*, analysis of the testicular weight *versus* body weight ratio between the sham side and the removal side at first week to fifth week after unilateral lipectomy in 8-week-old mice, n = 4. *D*, analysis of the epididymal weight *versus* body weight ratio between the sham side and the removal side at first week to fifth week after unilateral lipectomy in 8-week-old mice, n = 4. *E*, representative images of ovaries in 5-week-old mice with unilateral removal surgery at the second week to the fourth week. *F*, analysis of the ovarian weight *versus* body weight ratio at the first week to the fourth week after unilateral lipectomy in 5-week-old mice, n = 4. ∗*p* < 0.05, ∗∗∗*p* < 0.001. gWAT, gonadal white adipose tissue.

We performed unilateral lipectomy or bilateral lipectomy to determine the effect of gWAT on the local gonadal microenvironment through comparisons of testes or ovaries of both sides (Fig. 1*A*). First, we determined the testis size at different times after unilateral lipectomy and found that the testicular mass on the removal side was significantly increased at the second week and then returned to the normal level at the fourth week after unilateral lipectomy (Fig. 1, *B* and *C*). The epididymal mass of the removal side decreased at both the first to fifth weeks after unilateral lipectomy in 8-week-old mice (Fig. 1, *B* and *D*). We also performed lipectomy on mice of 4 weeks (prepuberty), 8 weeks (adolescent), and 16 weeks (adult) and measured testicular and epididymal mass at second and fourth weeks after unilateral lipectomy; it showed that the phenotypes of mice of different ages are consistent, which may indicate that the abnormal spermatogenesis caused by adipose loss is not affected by the testis developmental stage (Fig. S1, *A* and *B*). And the testis and epididymis size of sham side in unilateral lipectomy mice were also similar to that of unprocessed C57BL6 mice (control) at the same age (Fig. S1, *C* and *D*). All this proved that the unilateral lipectomy did not affect the body homeostasis of the mice. Similar to the phenotype in males, we found that the ovary of the unilateral removal side was significantly smaller than that of the sham side in female mice (Fig. 1, *E* and *F*). And the mass of ovary in bilateral pWAT removal mice was also decreased compared with bilateral sham mice. Overall, pWAT loss resulted in decreased ovarian weight. Thus, our data indicated that unilateral lipectomy might not affect body homeostasis, as the size of the contralateral gonad did not change but altered the size of the adjacent gonad.

### Unilateral lipectomy in male affects spermatogenesis in adjacent testis

**Figure 2:**
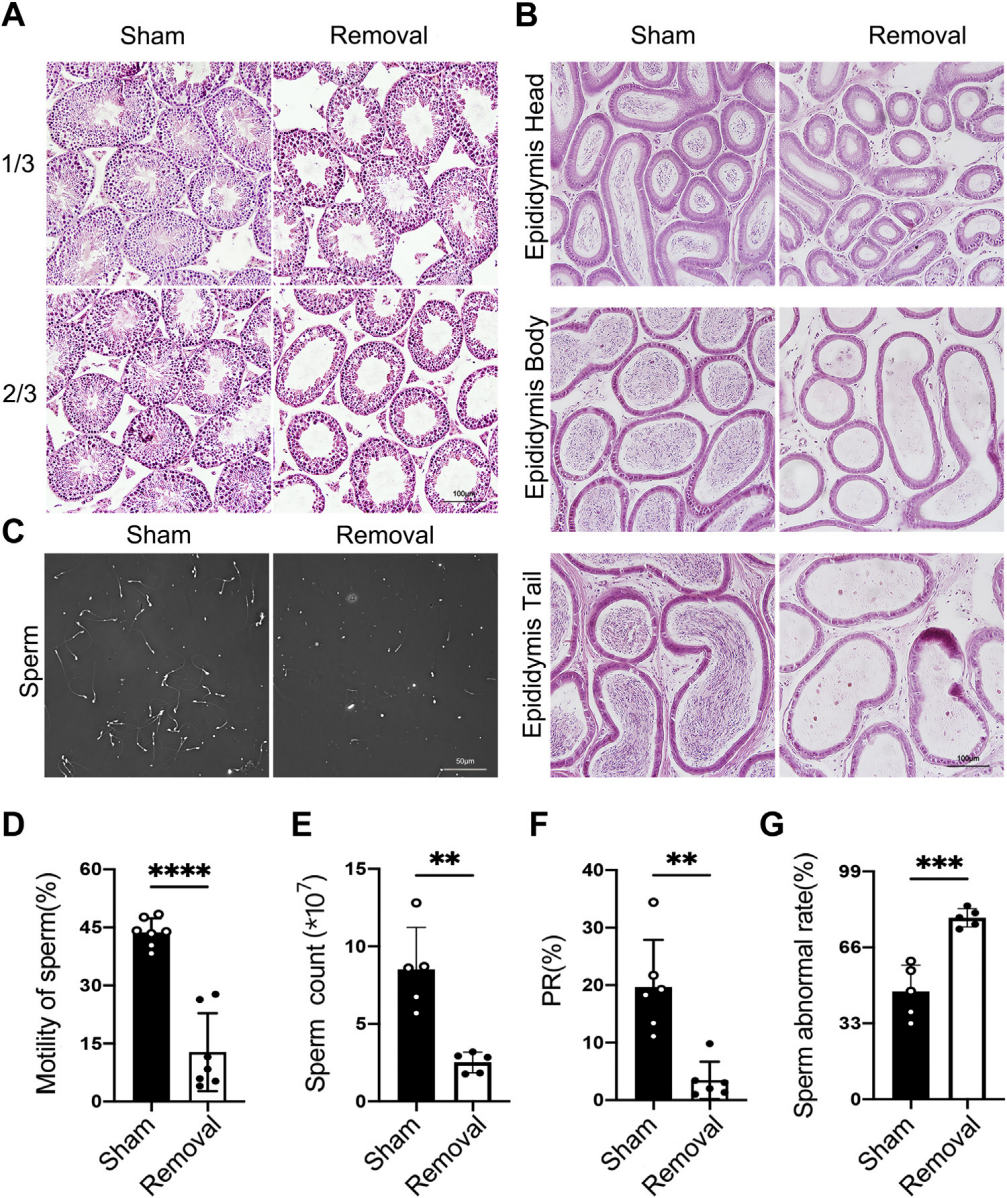
**The seminiferous tubule cellular lining thickness, sperm counts, and sperm quality were significantly decreased in mice that underwent epididymal adipose lipectomy.***A*, H&E staining of testis of the sham side and the removal side at the fourth week after partial unilateral lipectomy in 8-week-old mice; 1/3, remove a third of the eWAT; 2/3, remove two-thirds of the eWAT. *B*, H&E staining of the epididymis of the sham side and removal side at the fourth week after unilateral lipectomy in 8-week-old mice. *C*, representative photographs of the sperm from the sham side and removal side at the fourth week after unilateral lipectomy in 8-week-old mice. *D*, sperm motility analysis of the sham side and removal side at the fourth week after unilateral lipectomy in 8-week-old mice by CASA analysis, n = 7. *E*, sperm count analysis of the sham side and removal side at the fourth week after unilateral lipectomy in 8-week-old mice, n = 5. *F,* sperm PR analysis of  the sham side and removal side at the fourth week after unilateral lipectomy in 8-week-old mice by CASA analysis , n = 6. *G*, analysis of the abnormal sperm rate of the sham side and removal side at the fourth week after unilateral lipectomy in 8-week-old mice, n = 5. ∗∗*p* < 0.01, ∗∗∗*p* < 0.001, and ∗∗∗∗*p* < 0.0001. CASA, computer-assisted semen analysis; eWAT, epididymal white adipose tissue; PR, progressive movement.

To examine the effect of unilateral lipectomy on spermatogenesis, we performed H&E staining of testes after unilateral lipectomy. In different-aged mice at second and fourth weeks after unilateral lipectomy, the results showed that the thickness of the seminiferous tubule of removal side was significantly decreased compared with that of sham side (Fig. S2, *A* and *B*). The decrease in seminiferous tubule thickness was correlated to the mass of eWAT removed because one-third and two-thirds of adipose removed reduced the seminiferous tubule thickness to varying degrees (Figs. 2*A* and S2*C*). Abnormal spermatogenesis led to the absence of mature sperm in epididymis. Few sperms were observed in the head, body, and tail of the epididymis (Fig. 2*B*). Computer-assisted semen analysis also showed that the sperm counts, motility, and progressive rate were decreased (Fig. 2, *C*–*F*), whereas the rate of abnormal sperm significantly increased (Fig. 2*G*). These data suggested that eWAT was important for spermatogenesis and that a decrease in adipose mass could affect sperm quality. Chu *et al.* (13) reported that circulating concentrations of LH and testosterone (T) were not significantly different in lipectomy hamsters compared with the sham groups. Our result also proved that the deficiency of eWAT had no effect on testicular T level (Fig. S2*D*). These data suggested that unilateral lipectomy only affects spermatogenesis in adjacent testis.

### Unilateral lipectomy in female affects follicle development in adjacent ovary

**Figure 3:**
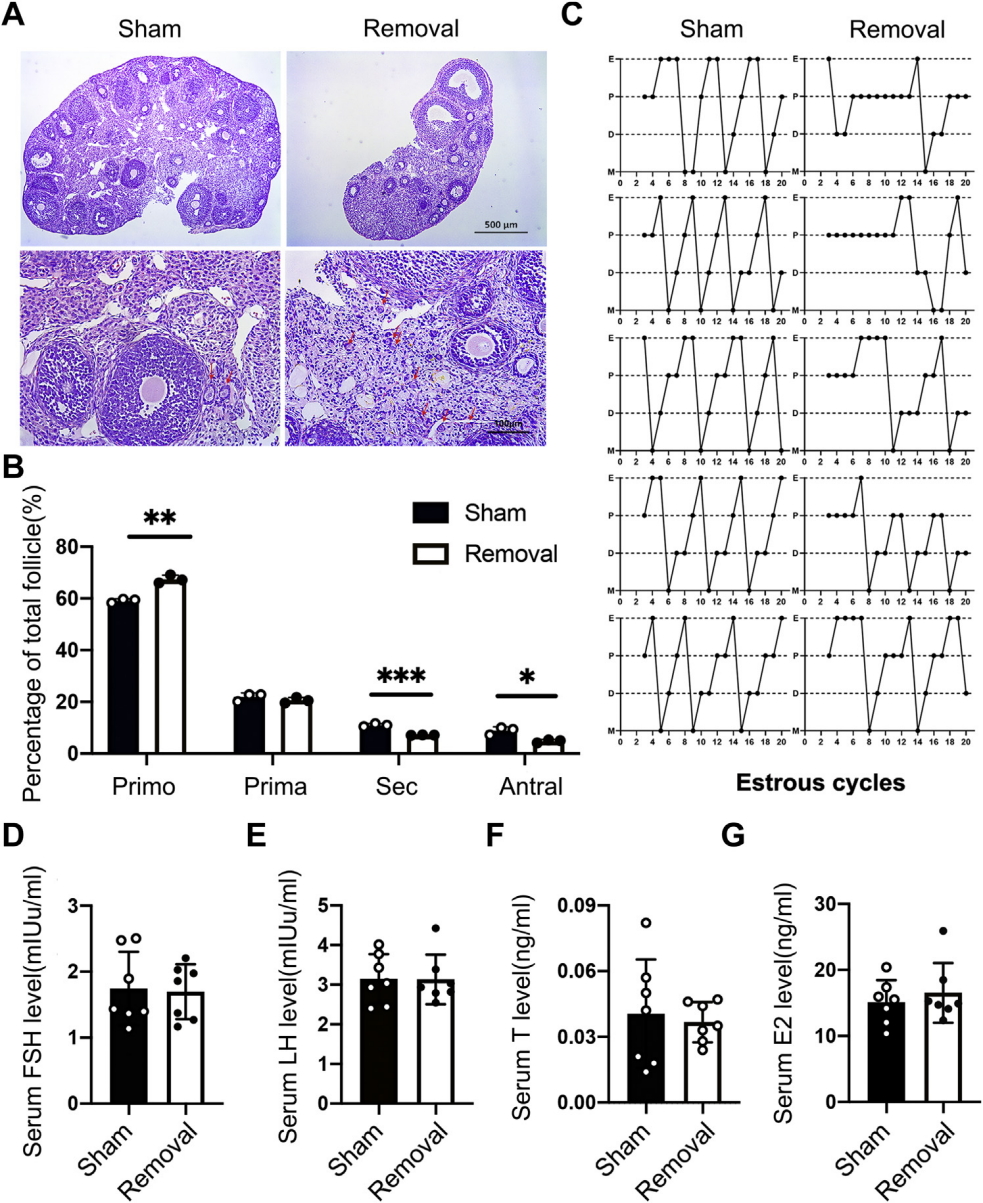
**Periovarian adipose tissue is important to follicle development, but it does not work by affecting hormone levels.***A*, H&E staining of ovaries of the sham side and removal side at the fourth weeks after unilateral lipectomy in 5-week-old mice. *B*, analysis of the number of follicles at different stages at the fourth week after unilateral lipectomy in 5-week-old mice, ∗*p* < 0.05, ∗∗*p* < 0.01, and ∗∗∗*p* < 0.001, n = 5. *C*, vaginal smear assays were conducted daily to monitor estrous cycles after lipectomy and sham surgery in 5-week-old mice. The *graph* shows representative cycles from the two groups. Each *box* indicates one animal, and *dots* represent a day; n = 5. *D* and *E*, analysis of serum concentrations of FSH and LH lever as measured by RIA in 5-week-old female mice after pWAT_ unilateral lipectomy and sham surgery, n = 7. *F*, analysis of serum concentrations of T lever as measured by RIA in 5-week-old female mice after pWAT unilateral lipectomy and sham surgery, n = 7. *G*, analysis of serum concentrations of E2 lever as measured by RIA in 5-week-old female mice after pWAT unilateral lipectomy and sham surgery, n = 7. D, diestrus; E, estrus; E2, estradiol 2; FSH, follicle-stimulating hormone; LH, luteinizing hormone; M, metestrus; P, proestrus; pWAT, periovarian white adipose tissue; RIA, radioimmunoassay; T, testosterone.

To examine the effect of unilateral lipectomy on follicle development, we performed H&E staining of ovary after lipectomy. We found that both unilateral lipectomy and bilateral lipectomy had severe effects on follicle development at second and fourth weeks after surgery, and the corpus luteum was barely observed in the ovary (Fig. 3*A* and Fig. S2, *E* and *F*).

The activation of primordial follicles was inhibited with the number of primordial follicles in the ovary that increased significantly, whereas the growing follicles were decreased in removal group, especially secondary follicles and antral follicles (Fig. 3, *A* and *B*). Female mice have four estrus cycles: proestrus, estrus, metestrus, and diestrus. Generally, they stay for 1 day in each period (20). In the 5-week-old mouse model, adipose removed leaded to disorder of the estrus cycle in mice, and estrous cycles were monitored daily during this period while the mice from the sham group exhibited regular estrous cycles of 4 to 5 days of duration (Fig. 3*C*). These data suggested that pWAT is important for follicle development. In order to examine the whole-body hemostasis in this process, we tested the concentrations of FSH, LH, T, and E2 in female mice. Although the serum FSH and LH levels were changed in the bilateral lipectomy mice compared with the sham mice (14), circulating concentrations of FSH, LH, T, and E2 did not change in our unilateral lipectomy mice comparing with sham mice (Fig. 3, *D*–*G*). These data suggested that unilateral lipectomy in female only affects follicle development in adjacent ovary.

### Lipectomy increases apoptosis and blocks proliferation in seminiferous tubules

**Figure 4:**
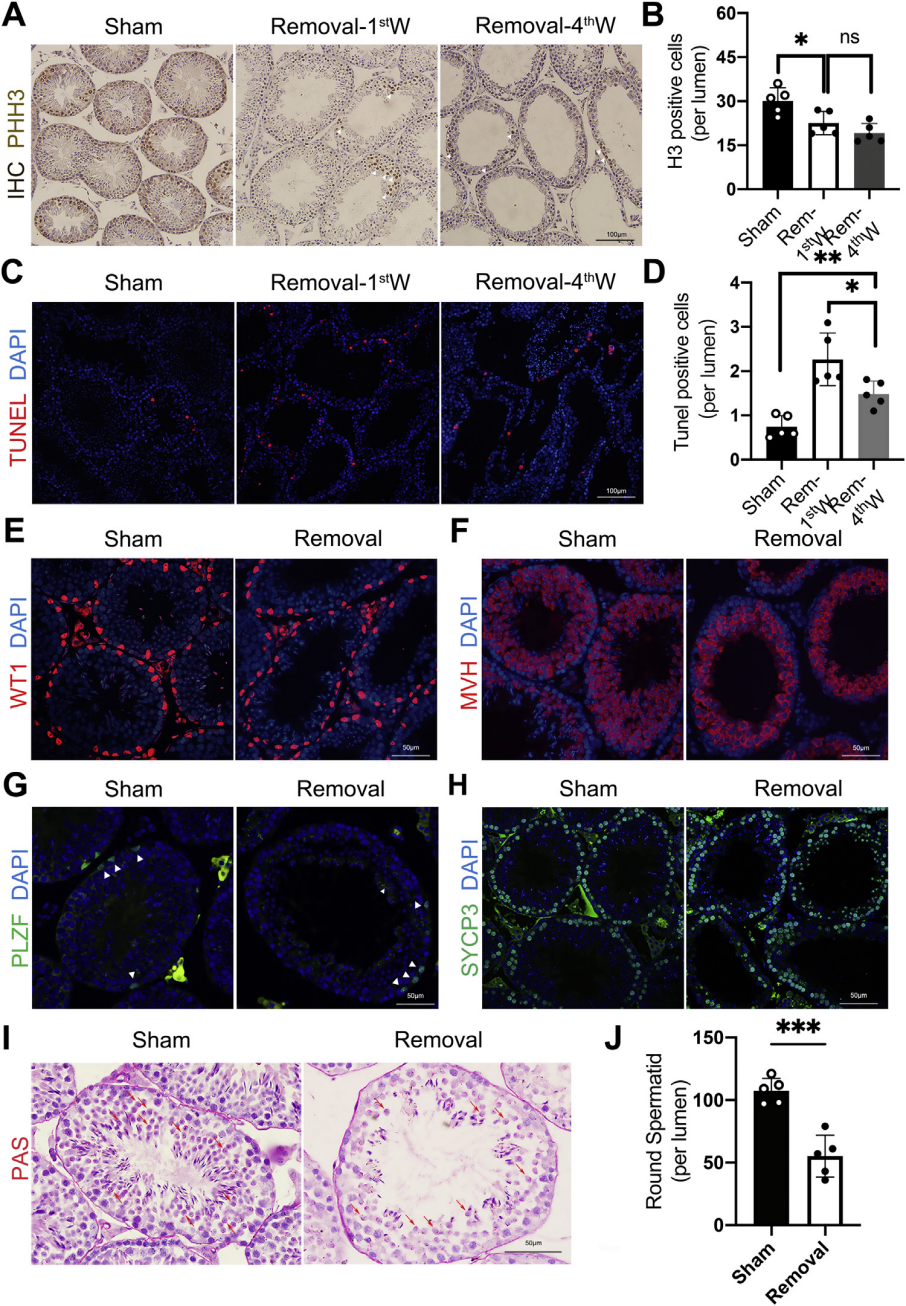
**Epididymal adipose lipectomy mainly results in loss of spermatids and increased apoptosis of germ cells in seminiferous tubules.***A*, immunohistochemistry staining of proliferating marker PHH3 in testes of the sham side and removal side at the first week and fourth week after unilateral lipectomy. *B*, analysis of PHH3-positive cell number in testes of the sham side and removal side at the first week and fourth week after unilateral lipectomy, n = 5. *C*, TUNEL apoptosis detection of cell in testes of the sham side and removal side. *D*, analysis of TUNEL-positive cell number in testes of the sham side and removal side at the first week and fourth week after unilateral lipectomy, n = 5. *E*, immunofluorescence staining of the SC marker WT1 in testes of the sham side and removal side at the fourth week after unilateral lipectomy. *F*, immunofluorescence staining of the GC marker mouse vasa homolog (MVG) in testes of the sham side and removal side. *G*, immunofluorescence staining of the spermatogonia stem cells marker Plzf in testes of the sham side and removal side at the fourth week after unilateral lipectomy. *H*, immunofluorescence staining of the spermatocyte marker Sycp3 in testes of the sham side and removal side at the fourth week after unilateral lipectomy. *I*, PAS staining of acrosome. PAS-positive substances (glycogen or polysaccharide) are *red*. *Red arrow* points to the round spermatids. *J*, analysis of round spermatid number in testes of the sham side and removal side at the fourth week after unilateral lipectomy, n = 5. ∗*p* < 0.05, ∗∗*p* < 0.01, and ∗∗∗*p* < 0.001. GC, germ cell; PAS, periodic acid–Schiff; PHH3, phosphohistone H3; SC, Sertoli cell.

Our data indicated that seminiferous tubule thickness was decreased after lipectomy. To determine the reason for this, we first performed proliferation assay and apoptosis analysis. Phosphohistone H3 staining and TUNEL reaction indicated that proliferation was inhibited (Fig. 4, *A* and *B*), whereas the apoptosis was largely enhanced in unilateral lipectomy mice (Fig. 4, *C* and *D*). We examined somatic cells and germ cells in the seminiferous tubules. Immunofluorescence staining of the Sertoli cell marker WT1 showed that unilateral lipectomy did not affect the counts of Sertoli cells in the seminiferous tubule (Figs. 4*E* and S3*A*). While the number of germ cells was largely decreased, as revealed by mouse vasa homolog staining (Figs. 4*F* and S3*B*). However, the deficiency of eWAT did not lead to the reduction of spermatogonial stem cells in testis detected by the marker Plzf (Figs. 4*G* and S3*C*). Further examination of Sycp3 immunofluorescence staining indicated that there was also no meiosis arrest (Figs. 4*H* and S3*D*). The decrease of germ cell population was because of the significant decrease of round spermatids revealed by periodic acid–Schiff staining (Fig. 4, *I* and *J*). We also counted the number of different types of cells in the testis of different-aged mice according to the histological morphology, and the conclusion was the same as the aforementioned immunostaining results (Fig. S4, *A*–*H*). In brief, epididymal adipose lipectomy blocked cell proliferation and augmented apoptosis in the seminiferous tubules that may be one of the causes of abnormal spermatogenesis.

### Gonadal adipose removal changes the metabolic and immune niches in testes and ovaries

**Figure 5:**
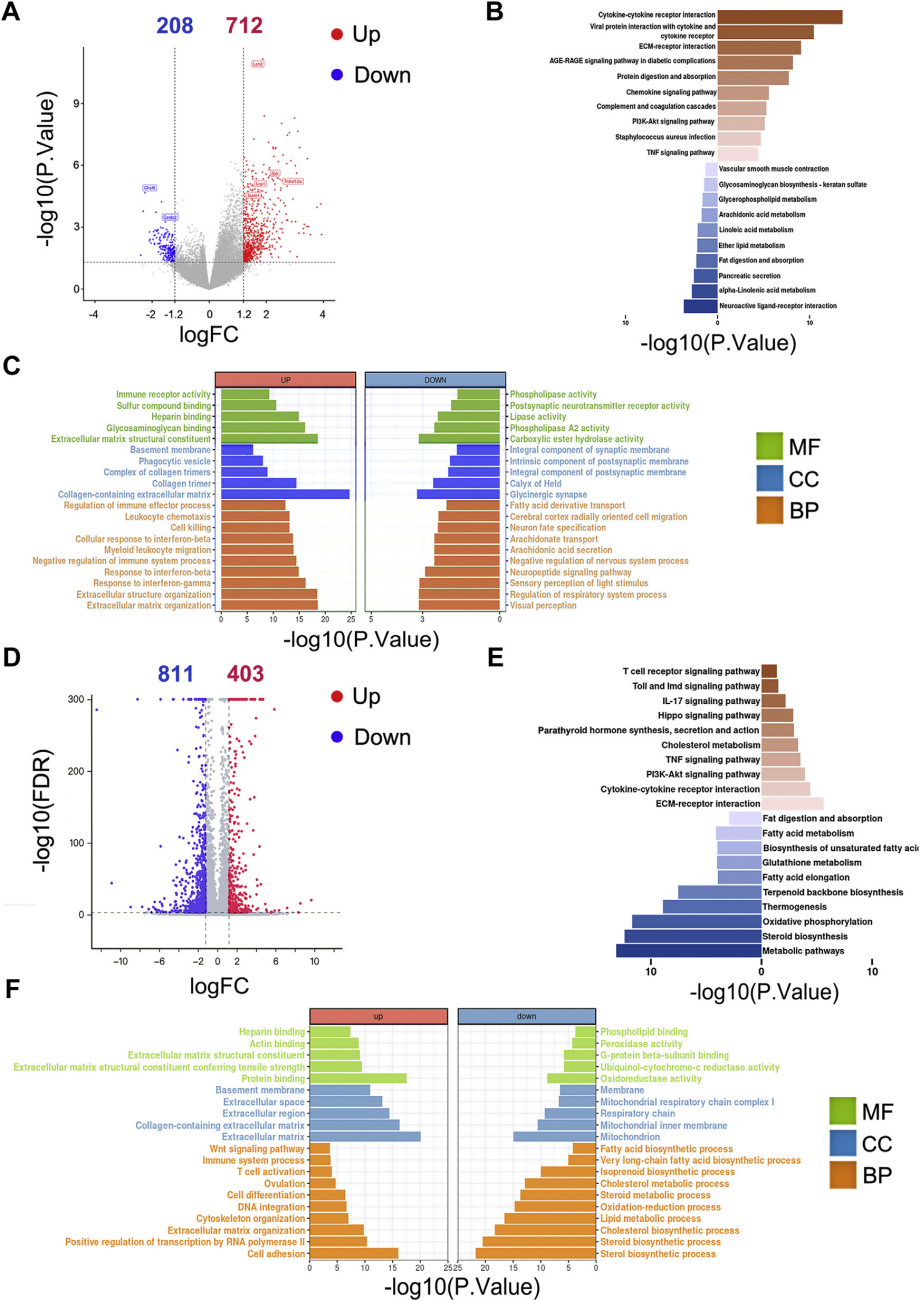
**Unilateral lipectomy damages the immune and metabolic local microenvironment of testes and ovaries.***A*, volcano plot showing the number of transcripts with upregulated and downregulated expression between the sham and removal groups in testes. Log2 fold change ≥1.2. *B*, results of KEGG enrichment analysis of the genes with upregulated and downregulated expression between the sham and removal groups in testes. *p* < 0.05. *C*, results of GO enrichment analysis of the genes with upregulated and downregulated expression between the sham and removal groups in testes. *p* < 0.05. *D*, volcano plot showing the number of transcripts with upregulated and downregulated expression between the sham and removal groups in ovaries. Log2 fold change ≥1.2. *E*, results of KEGG enrichment analysis of the genes with upregulated and downregulated expression between the sham and removal groups in ovaries. *p* < 0.05. *F*, results of GO enrichment analysis of the genes with upregulated and downregulated expression between the sham and removal groups in ovaries. *p* < 0.05. GO, Gene Ontology; KEGG, Kyoto Encyclopedia of Genes and Genomes.

To further reveal the working mechanism of gonadal adipose tissue in the testis and ovary, we performed transcriptomic analysis of testes at second and fourth weeks after unilateral lipectomy in 8-week-old mice. There were 712 annotated genes with upregulated expression and 208 with downregulated expression in the testis of the eWAT removal si0de compared with the sham side at second week after unilateral lipectomy (Fig. 5*A*). For Kyoto Encyclopedia of Genes and Genomes pathway analysis, the genes with upregulated expression were also found to be enriched in immune response pathways such as cytokine–cytokine receptor interaction, extracellular matrix–receptor interaction, and complement and coagulation cascade pathway. Moreover, the genes with downregulated expression were involved in metabolic pathways such as fat digestion and absorption, pancreatic secretion, and ether lipid metabolism (Fig. 5*B*). Gene Ontology analysis of differentially expressed genes showed that the genes with upregulated expression in the removal group were mainly enriched in categories related to extracellular matrix organization, immune response, and leukocyte migration. In contrast, genes with downregulated expression mainly participated in biological processes, such as neuropeptide signaling pathway, fatty acid derivative transport, and arachidonate transport (Fig. 5*C*).

After 4 weeks of postoperative recovery, the number of altered genes we detected in the testicles was less than that of second week after surgery. There were 327 annotated genes with upregulated expression and 107 annotated genes with downregulated expression in the testis of the eWAT removal side compared with the sham side at fourth week after unilateral lipectomy (Fig. S5, *A* and *B*). However, consistent with the aforementioned test results, in the testis at the fourth week after surgery, there are still upregulation of immune pathways and downregulation of metabolic pathways (Fig. S5, *C* and *D*). The transcriptomic data in the pWAT-removed ovaries also showed similar alterations to testis in both metabolic pathways and immune response pathways. We found that 403 annotated genes had upregulated expression and 811 annotated genes had downregulated expression in the whole pWAT-removed ovaries (Fig. 5*D*). The Kyoto Encyclopedia of Genes and Genomes analysis of genes with upregulated expression also mainly identified immune pathways, such as TNF signaling pathway and interleukin-17 signaling pathway. The genes with downregulated expression participated in steroid biosynthesis, fat digestion and absorption, metabolic pathway, and cholesterol metabolism (Fig. 5*E*). By Gene Ontology analysis, the differential genes in the ovary were mainly related to cytoskeleton organization, lipid metabolic process, cholesterol biosynthetic process, and sterol biosynthetic process (Fig. 5*F*). These data indicated that gWAT is important for the maintenance of metabolic and immune niches in both the male and female reproductive systems.

### Gonadal adipose maintains testis immune niches and blood–testis barrier integrity

**Figure 6:**
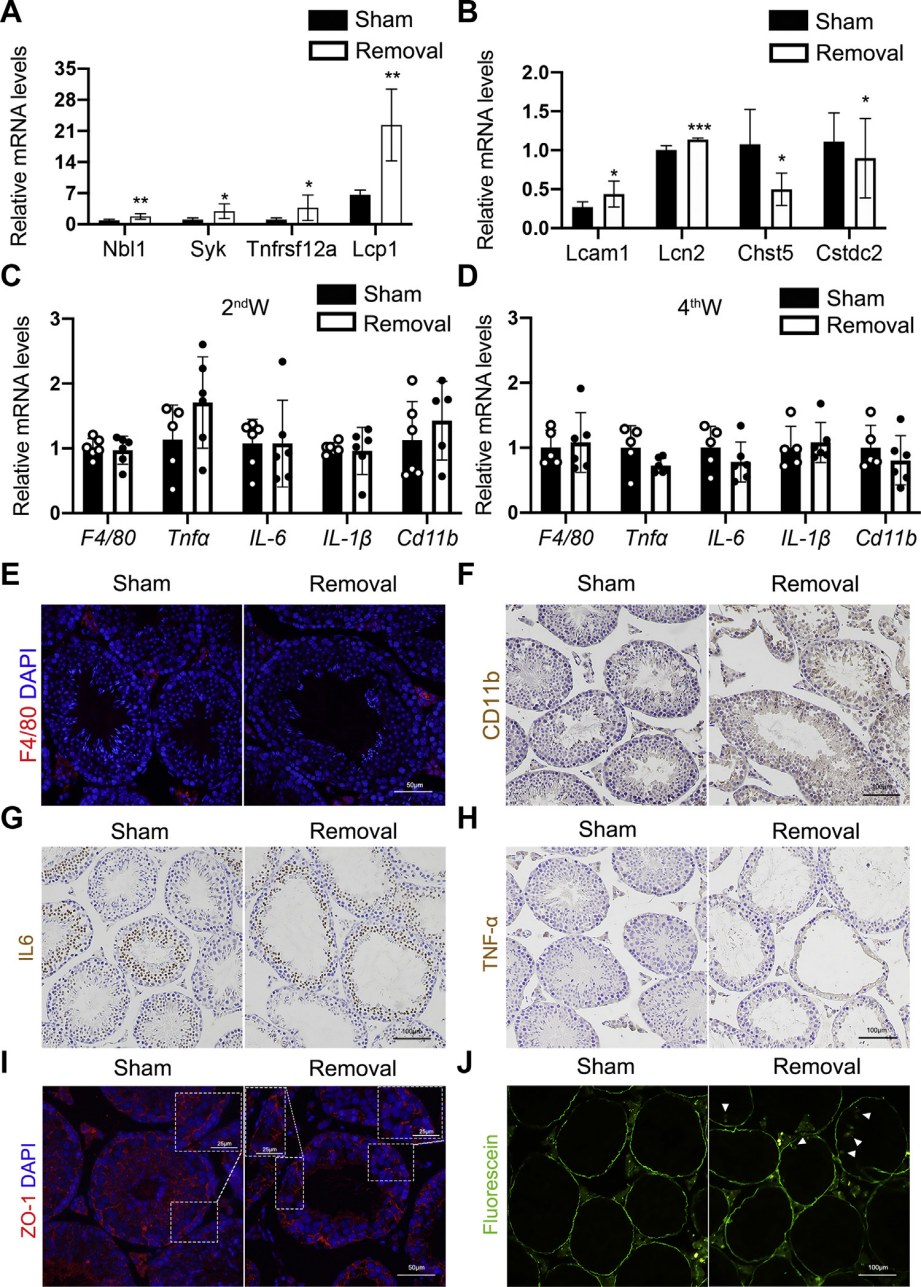
**Unilateral lipectomy altered immune environment and BTB integrity but not because of nonspecific damage.***A* and *B*, verification experiment of qPCR analysis of eight differentially expressed genes between the sham and removal groups. n = 6. *C*, the expression of inflammation-related genes in the testis at the second week after lipectomy by RT–qPCR analysis. n = 6. *D*, the expression of inflammation-related genes in the testis at the fourth week after lipectomy by RT–qPCR analysis. n = 6. *E*, immunofluorescence staining of the mouse major macrophage marker F4/80 in testes from the sham side and removal side at the fourth week after unilateral lipectomy. *F*, immunohistochemistry staining of the mouse major macrophage marker CD11b in testes from the sham side and removal side at the fourth week after unilateral lipectomy. *G*, immunohistochemistry staining of the inflammatory factor IL6 in testes from the sham side and removal side at the fourth week after unilateral lipectomy. *H*, immunohistochemistry staining of the inflammatory factor TNF-α in testes from the sham side and removal side at the fourth week after unilateral lipectomy. *I*, immunofluorescence staining of the BTB structure marker ZO-1 in testes from the sham side and removal side at the fourth week after unilateral lipectomy in 8-week-old mice. *J*, fluorescence distribution in sham and removal mice testis after biotin injection in 8-week-old mice. The *asterisk* indicates the fluorescence penetrating into seminiferous tubules. ∗*p* < 0.05, ∗∗*p* < 0.01, and ∗∗∗*p* < 0.001. BTB, blood–testis barrier; IL6, interleukin 6; qPCR, quantitative PCR; TNF-α, tumor necrosis factor-α.

Dependent on our transcriptome data, we further examined the expression of eight selected genes as validation experiment, and the expression of these genes is consistent with the transcriptome results. Some of these genes, such as *Lcn2* (lipocalin-2) and *Syk* (spleen tyrosine kinase), are associated with immune responses (Fig. 6, *A* and *B*). Interestingly, although we found that the immune microenvironment of the testis was altered. But inflammatory factors did not change by quantitative PCR (qPCR) analysis, which is the same conclusion we got in the transcriptome (Fig. 6, *C* and *D*). F4/80 and CD11b staining also showed that there was no macrophage infiltration in the testis of removal group (Fig. 6, *E* and *F*). Furthermore, there was no significant difference of expression of inflammatory factors such as interleukin 6, and TNF-α in testis tissue of sham and removal groups revealed by immunostaining (Fig. 6, *G* and *H*). Although these confirmation data indicated that gonadal adipose removal did not significantly affect the inflammatory-related gene expression, we did find that the physical niche blood–testis barrier (BTB) was destroyed and that the organization of tight junction protein ZO-1 between Sertoli cells was broken (Fig. 6*I*). The fluorescence leakage experiment also indicated that BTB integrity was damaged (Fig. 6*J*). Our data suggested that gonadal adipose plays important role in maintenance of testis immune niches and BTB integrity, and this transition is not because of nonspecific damage.

### Gonadal adipose maintains testis glycerophospholipid metabolic homeostasis and spermatogenesis

**Figure 7:**
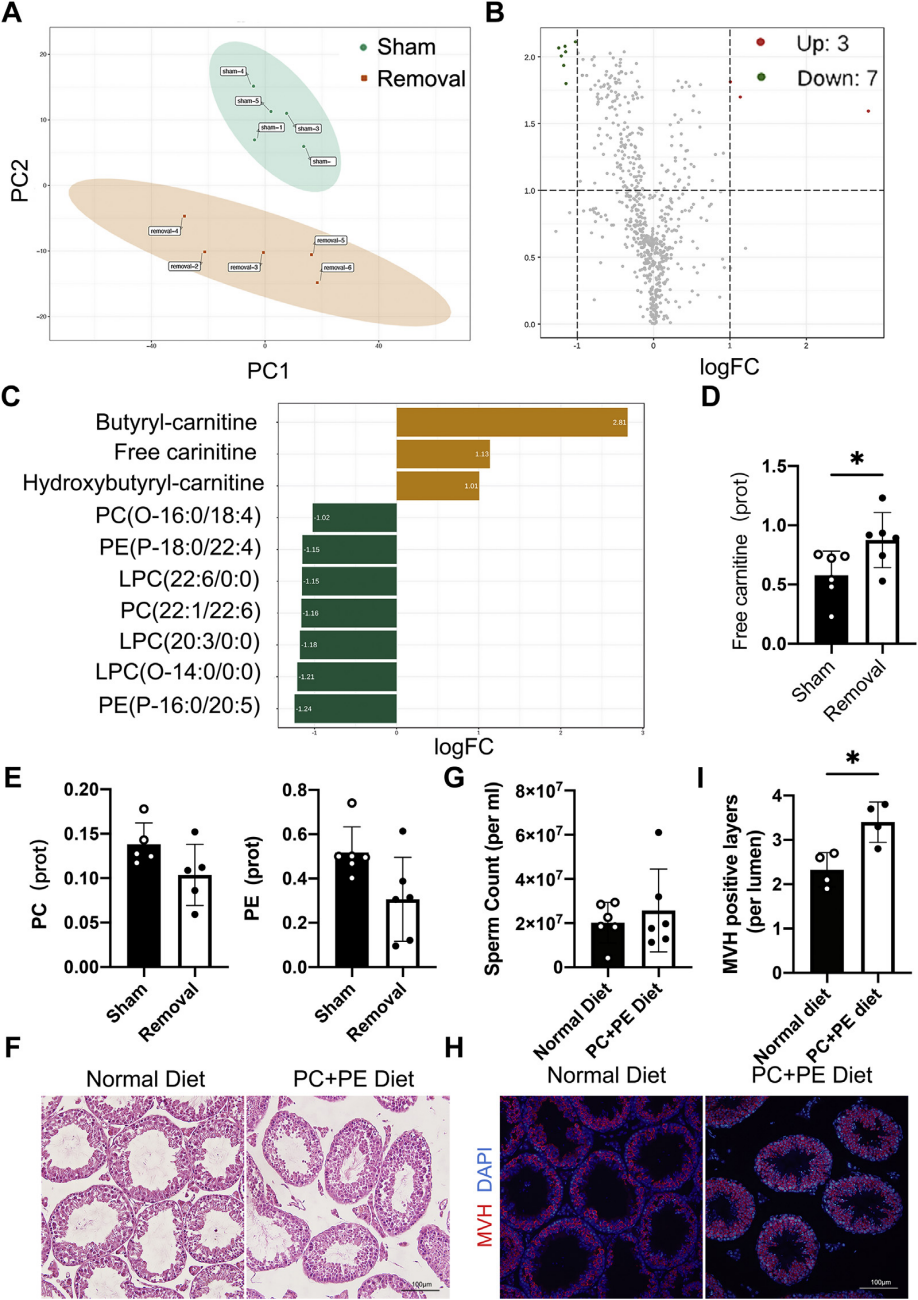
**Unilateral lipectomy alters the metabolic niche.***A*, PCA showing the distinction between the sham group and removal group. n = 5. *B*, volcano plot showing the increased and decreased lipid metabolites between the sham and removal groups. Fold change ≥1. *C*, details of 10 different lipid metabolites. *D*, verification of ELISA analysis of free carnitine, n = 6. *E*, verification of ELISA analysis of PC and PE, n ≥ 5. *F*, H&E staining of testis from 8-week-old lipectomy mice of PC + PE diet and normal diet at the fourth week after unilateral lipectomy. *G*, sperm count analysis of the sperm from 8-week-aged lipectomy mice of PC + PE diet and normal diet at the fourth week after unilateral lipectomy, n = 5. *H*, immunofluorescence staining of the GC marker MVH in testes from 8-week-aged lipectomy mice of PC + PE diet and normal diet at the fourth week after unilateral lipectomy. *I*, statistical analysis of MVH-positive cells, n = 4. ∗*p* < 0.05. GC, germ cell; MVH, mouse vasa homolog; PC, phosphatidylcholine; PCA, principal component analysis; PE, phosphatidylethanolamine.

gWAT is a tissue for lipid deposition and supports gonad lipid metabolism when needed. We found that most of the genes with upregulated expression participated in lipid metabolism after lipectomy, and then, we examined lipid and lipid derivatives in the testis after eWAT removal by lipidomics analysis. We found that lipid metabolites were either increased or decreased after lipectomy (Fig. 7, *A* and *B*). The enhanced metabolites were free carnitine, butyryl-carnitine, and hydroxybutyryl-carnitine, and the decreased metabolites contained three kinds of lysophosphatidylcholines, two kinds of phosphatidylcholines (PCs), and two kinds of phosphatidylethanolamines (PEs) (Fig. 7*C*). The increase of free carnitine in the removal groups was validated in further experiment (Fig. 7*D*), whereas the contents of PC and PE in the removed group only had a decreasing trend without significant difference (Fig. 7*E*). We supplemented the diet with PC and PE to lipectomy mice, which only showed a partial rescue phenomenon in the testis (Fig. 7*F*), although sperm counts were not significantly different compared with the mice fed the normal diet (Fig. 7*G*). The extent of germ cell loss was reduced in lipectomy mice fed the supplemental diet (Fig. 7, *H* and *I*). We concluded that eWAT can maintain glycerophospholipid metabolic homeostasis in the testis. In conclusion, our aforementioned data indicated that gWAT is important for gonadal function by directly affecting local metabolic and immune niches, besides whole-body homeostasis.

## Discussion

Past research has indicated that excessive body fat significantly impairs gametogenesis in healthy individuals (21). For males, overweight has a negative impact on semen quality, which is usually manifested as poor semen parameters (22). For females, obesity leads to infertility and ovulation disorders in the ovary (23). Although overweight is harmful to health, our study and other studies provided strong evidence that extreme weight loss can also negatively affect the reproductive system (24). In this study, we showed that a decrease in eWAT in male mice resulted in a reduction in spermatogenesis in the testis and sperm count and motility in the epididymis. Moreover, the lack of pWAT led to unsuccessful follicle development in female mice. Therefore, proper fat reserves are very important for reproduction, especially gWAT.

Studies have shown that WAT contains many adipokines and immune cells, forming a complex network of metabolism and immunity and participating in the development of insulin resistance. These adipokines can act on neighboring gonads through autocrine or paracrine mechanisms; for example, leptin (25), adiponectin (26), and resistin (27) can regulate the synthesis and secretion of sex hormones or regulate carbohydrate and lipid metabolism in gonads. In addition, these adipokines promote the activation, proliferation, and secretion of inflammatory factors or participate in the immune process. It is generally believed that adipokines mainly affect gametogenesis by regulating systemic endocrine signaling, especially through the hypothalamic–pituitary–gonadal axis (28). However, apart from the influence of systemic endocrine factors on the reproductive system, we believe that gWAT in the local microenvironment of the gonads is also crucial. Our study established a unilateral gWAT removal model without damaging the blood supply or nerves of testis at the endocrine level of the central nervous system in mice, and we found that the testicular function of the sham side is normal, whereas the testis of the removal side has lesions because of the different metabolic and immune microenvironments.

The testis is a typical immune-privileged organ; on the one hand, the systemic immune response is suppressed in the testis to prevent self-antigens from inducing harmful immune responses (29). On the other hand, testicular tissue–specific cells have established an effective natural immune defense mechanism to resist invading pathogens (30). Loss of eWAT enhanced testicular immune system function and damaged the balance of the immunosuppressive microenvironment. However, this enhancement not only destroyed the immune-privileged mechanism but also made the testes extremely sensitive to foreign substances. BTB is composed of a variety of connections between adjacent Sertoli cells, including tight junctions, specialized junctions, and gap junctions (31). The complex connections of the BTB strictly restrict the passage of molecules and cells and provide a suitable microenvironment for spermatogenesis (32). BTB separates the seminiferous tubules by a basal compartment and an adluminal compartment. The spermatocytes in zygotene and leptotene stages are located in the basal compartment; pachytene spermatocytes, diplotene spermatocytes, secondary spermatocytes, round spermatids, and elongated spermatids are located in the adluminal compartment. By separating the testis into two parts, BTB can better protect the germ cells located in the adluminal compartment after meiosis from being attacked by the body's immune system, thereby establishing a special immune-privileged environment inside the testis (33). The destruction of the BTB, a component of testicular immunity, may be one of the causes of inhibited spermatogenesis in eWAT-removed testes. This change in the immune microenvironment also occurs in pWAT-deficient ovaries. The effect of gWAT loss on the gonadal immune microenvironment was not related to the surgery itself, as there was no severe inflammation in the testis and no changes in T levels within the testis.

In addition, a stable metabolic environment is very important for spermatogenesis. Studies have shown that low-fat diets can reduce male T levels by 10 to 15%, and vegetarians can reduce T levels by 26% (34). According to transcriptomic analysis, the genes with downregulated expression were mainly enriched in the lipid catabolic process, fat digestion or absorption, and fatty acid metabolic pathway, especially n-6 polyunsaturated fatty acid metabolism (arachidonic acid metabolism and linoleic acid metabolism), in the removal group. Polyunsaturated fatty acids mainly include n-6 polyunsaturated fatty acids and n-3 polyunsaturated fatty acids. n-6 polyunsaturated fatty acids are involved in regulating blood coagulation, promoting cell proliferation, and participating in the immune system. n-3 polyunsaturated fatty acids can lower blood pressure and lipids, resist cancer, and reduce chronic inflammation.

An imbalance in the ratio of n-6 polyunsaturated fatty acids and n-3 polyunsaturated fatty acids can lead to abnormal spermatogenesis (35). In our data, we found abnormalities in n-6 polyunsaturated fatty acid metabolism, which may cause an imbalance in the proportion of polyunsaturated fatty acids. We also found that the glycerophospholipid metabolic process was abnormal in the removal group, consistent with the downregulation of PC, PE, and lysophosphatidylcholine expression in the lipidomics analysis. Apart from constituting biological membranes, glycerophospholipids are also one of the components of bile and membrane surface–active substances and are involved in the recognition and signal transduction of proteins by cell membranes. In order to explore whether the disruption of glycerophospholipid metabolic pathway is the cause of abnormal spermatogenesis, we performed PC and PE replenishment experiments in mice after eWAT resection. It was found that the addition of PC and PC to the diet facilitated the recovery of testis in lipectomy mice, although this did not completely rescue the spermatogenic phenotype. This may also indicate that disruption of the glycerophospholipid pathway is only part of the cause of abnormal spermatogenesis, and changes in the immune microenvironment also play an important role in this process. In the ovaries, we found that a lack of pWAT affects the biosynthesis of steroids. Steroids mainly include various estrogens, such as E2 and progesterone. E2 can inhibit endoplasmic reticulum stress–related apoptosis and inflammation, regulate lipid metabolism, and exert cytoprotective effects (36, 37). Progesterone is secreted by the ovaries and can inhibit the development of primordial follicles and dominant follicles (38). We suspect that the disorder of steroid synthesis caused by a low-fat state is the main reason for abnormal ovulation.

In conclusion, low-fat status may present potential risks to reproductive dysfunction by disrupting the immune and metabolic microenvironments in the gonads, which may be related to the endocrine or paracrine functions of white adipose tissue. More research is needed to explore the mechanism by which gWAT affects the immune and metabolic microenvironments of the reproductive system.

## Experimental procedures

### Animals

Eight-week-old male and five-week-old female C57BL6 mice were purchased from GemPharmatech and then housed in accordance with regulations on mouse welfare and ethics of Nanjing University in groups with a 12 h light/12 h dark cycle, a room temperature of 21 °C, 50% humidity, and free access to food and water. All animal experiments were approved by the medical school of Nanjing University, Nanjing, China and performed according to the recommendations of the National Institutes of Health Guide for the Care and Use of Laboratory Animals.

### Lipectomy

After 7 days of adaptation to these housing conditions, lipectomy was performed on mice after inhalational anesthesia with isoflurane. For males, different treatments were performed on the left and right sides of the same mice, eWAT was removed on the right side, and sham surgery was performed on the left side. First, the mouse ventral region was disinfected with 75% ethanol after shaving, and a minimal incision was made to the ventral region adjacent to the leg through the skin and peritoneum. After careful exposure of the eWAT adipose tissue on the right side, generally speaking, the adipose tissue was removed without damaging the blood supply or nerves of the testis. The incision was subsequently closed with wound clips and absorbable surgical sutures. Sham surgery, which was used for the control group, included incision and exposure of the eWAT without removal on the left side. The treatment of female mice was the same as that of male mice, and the operating object of female mice was pWAT. In addition to the unilateral lipectomy model, female mice had a bilateral lipectomy model and bilateral sham model. The mouse was placed on a heating table after the operation to maintain body temperature and promote the recovery of the mouse.

### Morphology, histology, immunohistochemistry, immunofluorescence, and fluorescein permeability assay

To explore the morphology of the testes and ovaries after lipectomy, we sacrificed the mice at several different postoperative times. Fresh tissues were fixed overnight at 4 °C in 4% PBS-buffered paraformaldehyde for H&E, immunohistochemistry, and immunofluorescence staining, dehydrated in ethanol, embedded in paraffin, and cut by microtomes into 5 μm paraffin sections. For histological analysis, the sections were stained with H&E according to a standard protocol. For immunofluorescence staining, paraffin sections were deparaffinized, rehydrated, and boiled in citrate buffer (pH 6.0) for antigen retrieval. Afterward, paraffin sections were permeabilized, blocked, and incubated with the indicated primary antibodies at 4 °C overnight. Subsequently, the sections were incubated with fluorescent secondary antibodies for 1 h at room temperature and then stained with 4′,6-diamidino-2-phenylindole. For immunohistochemistry staining, paraffin sections were deparaffinized, rehydrated, incubated with hydrogen peroxide at 37 °C for 30 min, and boiled in citrate buffer (pH 6.0) for antigen retrieval. Afterward, paraffin sections were permeabilized, blocked, and incubated with the indicated primary antibodies at 4 °C overnight. Subsequently, the sections were incubated with secondary antibodies for 1 h at room temperature. 3,3′-Diaminobenzidine chromogenic reagent was incubated for 10 min and stained with hematoxylin. A fluorescein assay for BTB integrity was performed as previously described (39).

### Sperm motility, capacitation, and sperm abnormality rate analysis

The sperms from the epididymis were allowed to release and capacitate in 500 μl of human tubal fluid (HTF) medium. For sperm motility analysis, computer-assisted semen analysis was performed after sperm capacitated for approximately 10 min in a 37 °C thermostatic water bath. For sperm counting experiments, a small incision was made on the fresh epididymis tail, and the sperms were placed in 1 ml of HTF medium or PBS solution, moved to a 37 °C baking oven for 10 min, and counted using automated cell counters (ALIT Life Science Co, Ltd). For determination of the sperm abnormality rate, 10 μl sperm HTF suspension droplets were added to the slide and spread evenly. Then, the cells were fixed with methanol until natural drying, stained with Oil Red after 30 min of fixation, and then air dried. The ratio of abnormal spermatozoa was analyzed and counted under the microscope.

### Estrous cycle analysis

Vaginal smears were collected on glass slides in 10 μl of 0.9% NaCl at 07:00 to 08:00 h each morning. After air drying, samples were stained with Toluidine Blue O (Amresco) for 3 to 4 min and then washed and dried. The four stages of the estrous cycle were determined as previously described by analyzing the proportion of three major cell types (epithelial cells, cornified cells, and leukocytes) (20). Consistent cycles of proestrus, estrus, metestrus, and diestrus (4–5 days total) in mice were called “regular cycles.”

### Ovarian follicle counting

Ovaries were collected and fixed in 10% buffered formalin for 12 h, embedded in paraffin, serially sectioned at a thickness of 5 μm, and then stained with H&E. All follicles with a visible nucleus were counted every second section, and analysis was performed on every section. Follicle classification was determined as follows (40): oocytes surrounded by a single layer of flattened or cuboidal granulosa cells were defined as primordial and primary follicles; oocytes surrounded by more than one layer of cuboidal granulosa cells with no visible antrum were determined to be secondary follicles. Antral follicle possessed a clearly defined antral space and a cumulus granulosa cell layer. Corpora lutea were filled with lutein cells, and follicles were considered atretic if they contained either a degenerating oocyte, disorganized granulosa cells, pyknotic nuclei, shrunken granulosa cells, or apoptotic bodies. The results are reported as the number of follicles counted per ovary.

### Real-time qPCR

The mouse testes were carefully isolated and quickly frozen in liquid nitrogen. Total cellular RNA was isolated by using TRIzol (Invitrogen) as described in the manufacturer’s instructions. Complementary DNA (cDNA) was synthesized by PrimeScript RT Master Mix (TaKaRa) from 1 μg of total RNA isolated with TRIzol reagent. The resulting cDNA was diluted 1:10 in sterile water, and aliquots of 1 μl were used for qPCR. Primers for all kinds of genes were synthesized by Integrated DNA Technology (Invitrogen). The resulting material was then used for independent qPCR, which was carried out on an Applied Biosystems HT7300 Sequence Detector. Each cDNA sample was run in triplicate, and the mRNA concentration in each sample was calculated relative to the vehicle control by using the 2-CT analysis method.

### RNA-Seq

Total RNA was extracted from frozen testis and ovarian tissue from the first or second week after lipectomy. RNA-Seq analysis was performed by Berry Genomics. The original file in FastQ format was downloaded, and quality control was performed with FastQC. All the RNA-Seq datasets were mapped onto the genome for control spike-in RNAs and the mouse genome (GRCm38.p6) using Hisat2 (41)(version 2.2.0) to obtain files in SAM format. Genome sequence files (GRCm38.p6) and annotation files were downloaded from ENSEML. FeatureCounts (42) (version 2.0.2) was used to quantify mapped counts of mouse genes separately. For normalizing mouse gene expression, we estimated the size factors of every RNA-Seq dataset using the R package Limma for differential analysis and then used the package ClusterProfiler for enrichment analysis.

### Metabolite detection by lipidomics

The data acquisition instrument system mainly includes ultraperformance liquid chromatography (ExionLC AD) and tandem mass spectrometry (QTRAP). The liquid phase conditions mainly include the following: (1) chromatographic column: Thermo Accucore C30 column, i.d. 2.1 × 100 mm, 2.6 μm; (2) phase A was acetonitrile/water (60/40, v/v) (containing 0.1% formic acid, 10 mmol/l ammonium formate), and phase B was acetonitrile/isopropanol (10/90, v/v) (containing 0.1% formic acid and 10 mmol/l ammonium formate); (3) the mobile phase gradient was 0 min for A/B (80:20, v/v), 2 min (70:30, v/v), 4 min (40:60, v/v), 9 min (15:85, v/v), 14 min (10:90, v/v), 15.5 min (5:95, v/v), 17.3 min (5:95, v/v), 17.5 min (80:20, v/v), and 20 min (80:20, v/v); (4) the flow rate was 0.35 ml/min, the column temperature was 45 °C, the injection volume was 2 μl, the gradient flow rate was 0.4 ml/min, and the column temperature was 40 °C.

Mass spectrometry conditions were electrospray ionization, temperature 500 °C, mass spectrometry voltage 5500 V in positive ion mode, mass spectrometry voltage −4500 V in negative ion mode, ion source gas 1 45 psi, gas 2 55 psi, curtain gas (curtain gas, CUR) 35 psi, and collision-activated dissociation parameter set to medium. In the triple quadrupole analysis, each ion pair was scanned based on the optimized declustering potential and collision energy. The quality control samples were injected four times at the start to ensure system consistency.

### Replenishment experiment

About 8% PC and 8% PE were added to the diet of mice, the experimental group was fed the supplemented feed for 5 weeks continuously, and the control group was fed the normal feed. After 7 days of adaptation to these housing conditions, lipectomy was performed according to the aforementioned method, and the mice were killed for 4 weeks after the operation.

### Statistical analysis

Statistical analysis was performed using GraphPad Prism 9 (GraphPad Software, Inc). The statistical significance of differences between groups was analyzed by two-way ANOVA, followed by least significant difference post hoc test to assess individual differences. *p* Values <0.05 were considered significant.

## Data availability

All data are contained in the article.

## Supporting information

This article contains supporting information.

## Conflict of interest

The authors declare that they have no conflicts of interest with the contents of this article.
