# Supplementary material for: Gonadal white adipose tissue is important for gametogenesis in mice through maintenance of local metabolic and immune niches
Source: J Biol Chem. 2022 Mar 10;298(5):101818. doi: 10.1016/j.jbc.2022.101818 (PMC9052151; doi:10.1016/j.jbc.2022.101818)
Supplement: Supplemental Figures S1–S5 [file mmc1.docx]

**Supporting information**

**S1**

**
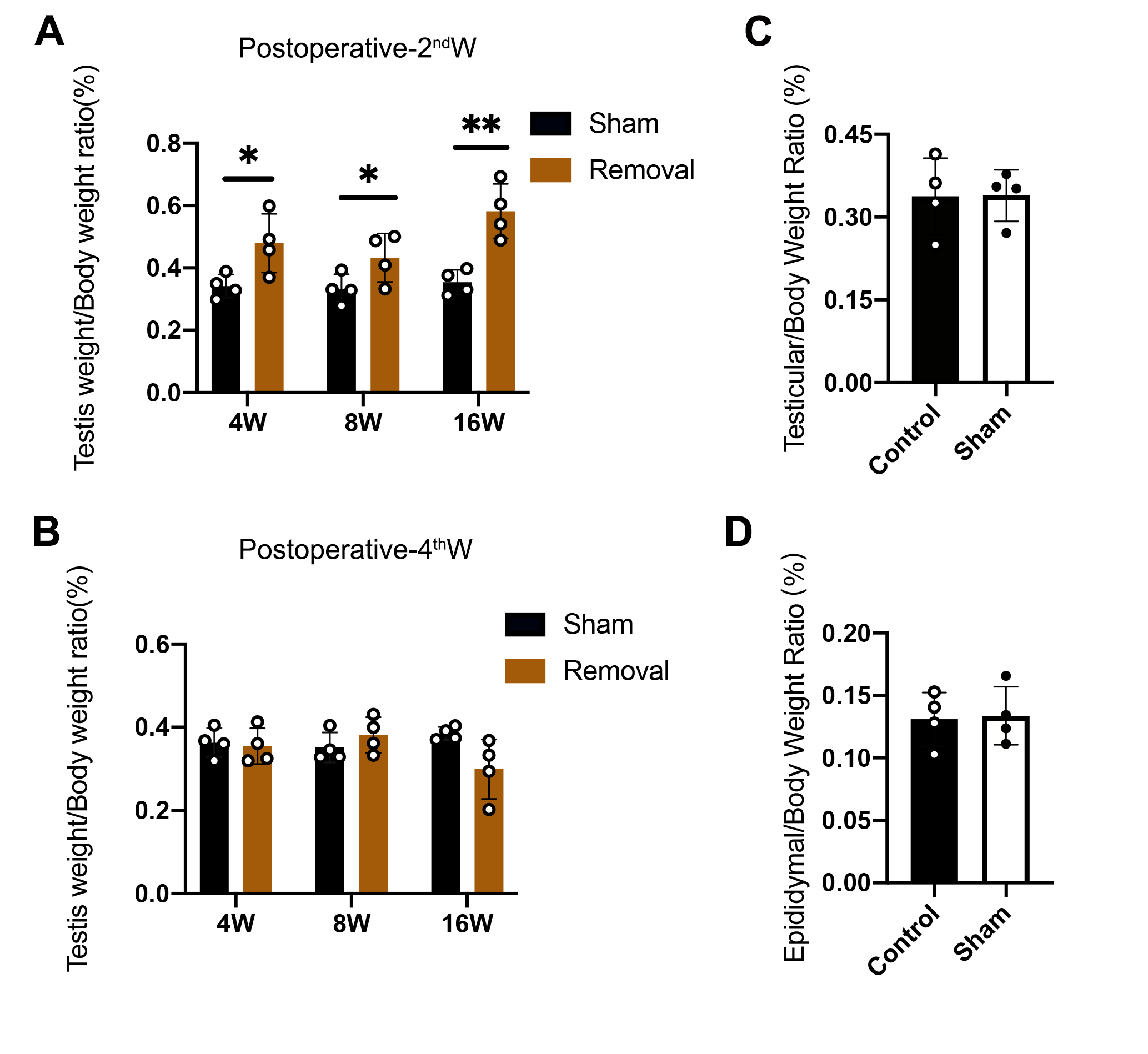
**

**S1 Fig. Deficiency of pWAT adipose caused the change of testicular and ovaries mass in mice.** **a b** Analysis of the testicular weight vs. body weight ratio between the sham side and the removal side in 4-week-old mice,8-week-old mice and 16-week-old mice at postoperative 2nd and 4th week, *n*=4. **c** Analysis of the testis weight vs. body weight ratio between the sham group (the testes of lipectomy mice sham side) and the control group (the testes of unprocessed C57BL6 mice). **d** Analysis of the epididymis weight vs. body weight ratio between the sham group (the epididymis of lipectomy mice sham side) and the control group (the epididymis of unprocessed C57BL6 mice).

**S2**


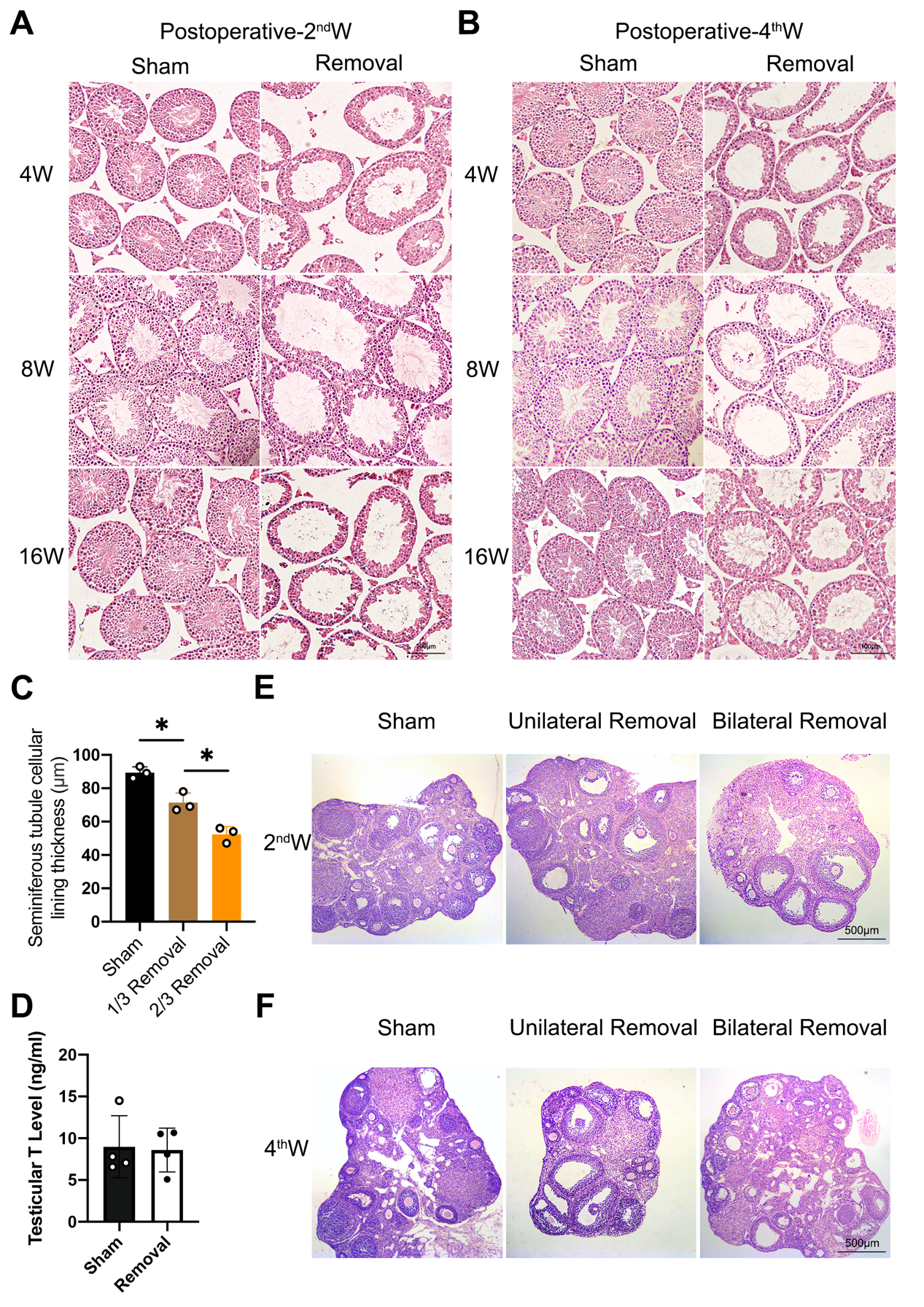


**S2 Fig. Lipectomy had a severe effect on spermatogenesis and folliculogenesis. a b** H&E staining of testis from the sham side and the removal side at the 2nd week and 4th week after unilateral lipectomy in 4-week-old mice,8-week-old mice and 16-week-old mice at postoperative 2nd and 4th week. Scale bar: 100 μm. **c** Analysis of the testis seminiferous tubule thickness at the 4th week after partial unilateral lipectomy between the sham and the 1/3 and 2/3 removal groups by ImageJ; 1/3, remove a third of the eWAT; 2/3, remove two-thirds of the eWAT. **p* < 0.05, *n*=3. **d** Analysis of Testicular concentrations of T as measured by RIA in male mice with sham surgery and unilateral lipectomy. *n*=4. **e f** H&E staining of ovaries on the right side from the bilateral sham group, unilateral removal group and bilateral removal group at the 2nd and 4th week after pWAT lipectomy. Scale bar: 500 μm.

**S3**


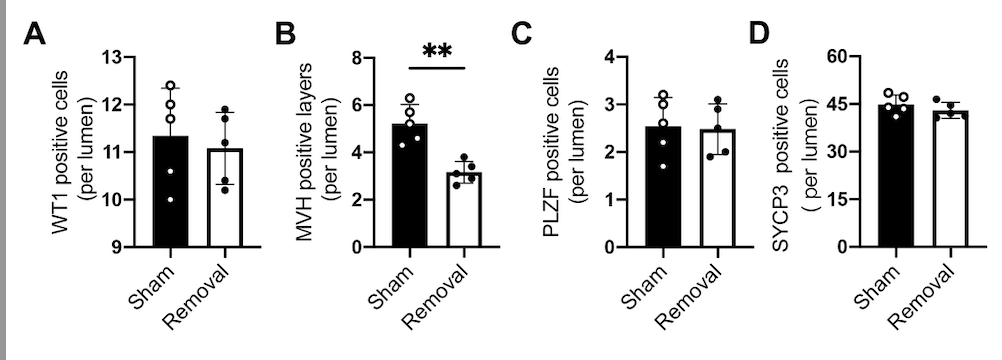


**S3 Fig. Epididymal adipose lipectomy mainly results in loss of germ cells in seminiferous tubules a** Analysis of WT1-positive cell number between sham group and removal group in 8-week-old mice at 4^th^ week after unilateral lipectomy. *n*=5. **b** Analysis of MVH-positive cell layers between sham group and removal group in 8-week-old mice at 4^th^ week after unilateral lipectomy. *n*=5. **c** Analysis of Plzf-positive cell number between sham group and removal group in 8-week-old mice at 4^th^ week after unilateral lipectomy. *n*=5. **d** Analysis of Sycp3-positive cell number between sham group and removal group in 8-week-old mice at 4^th^ week after unilateral lipectomy. *n*=5. ***p* < 0.01.

**S4**


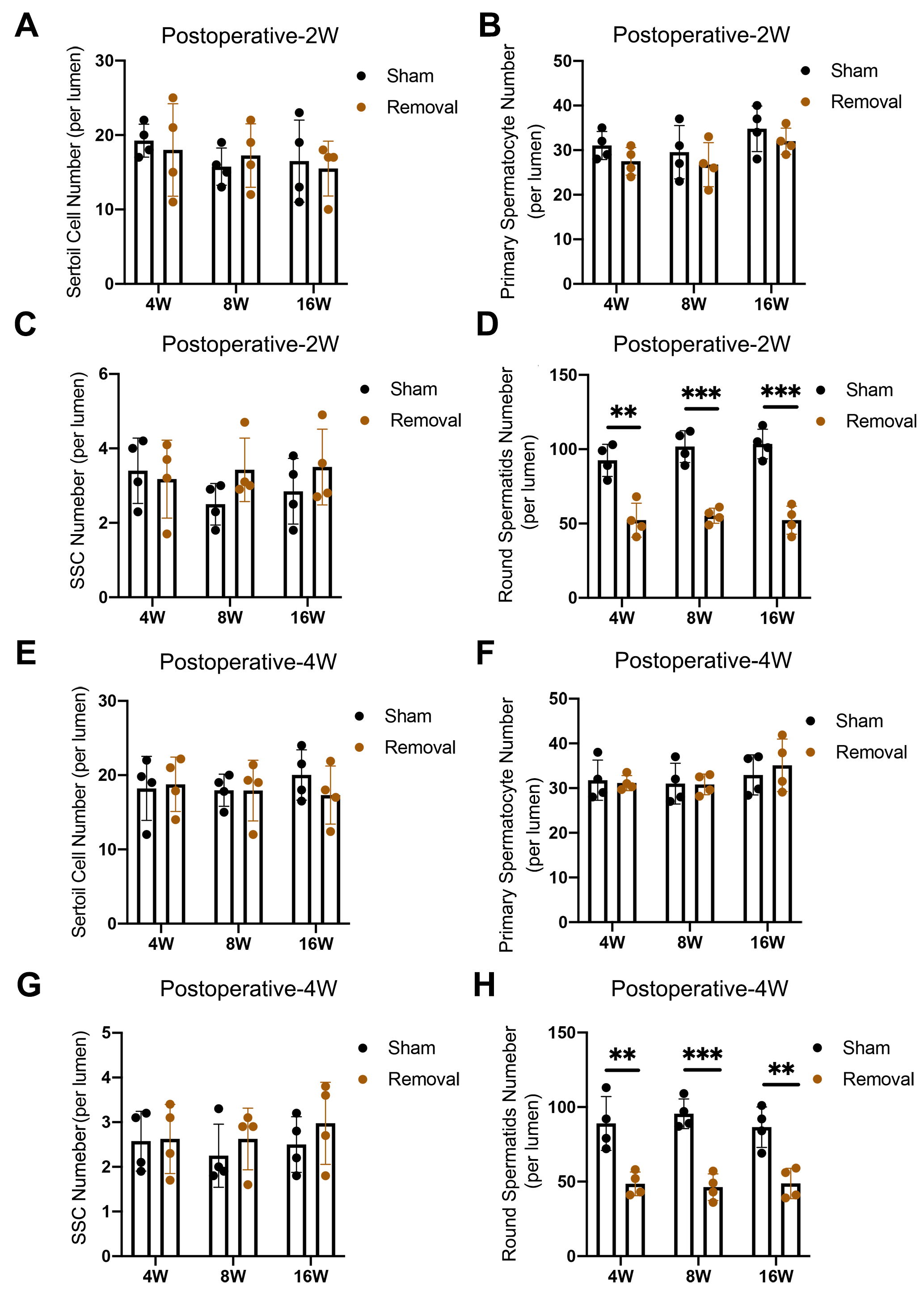


**S4 Fig. Analysis of different types of cells in seminiferous tubules of sham group and removal group. a** Analysis of Sertoil cell number of sham and removal groups at different ages of 2nd week after lipectomy. *n*=4.**b** Analysis of primary spermatocyte number of sham and removal groups at different ages of 2nd week after lipectomy. *n*=4. **c** Analysis of SSCs number of sham and removal groups at different ages of 2nd week after lipectomy. *n*=4. **d** Analysis of round sperm cell number of sham and removal groups at different ages of 2nd week after lipectomy, *n*=4. **e** Analysis of Sertoil cell number of sham and removal groups at different ages of 4th week after lipectomy. *n*=4.**f** Analysis of primary spermatocyte number of sham and removal groups at different ages of 4th week after lipectomy, *n*=4. **g** Analysis of SSCs number of sham and removal groups at different ages of 4th week after lipectomy. *n*=4.**h** Analysis of round sperm cell number of sham and removal groups at different ages of 4th week after lipectomy, **p* < 0.05, ***p* < 0.01, ****p* < 0.001. *n*=4.

**S5**


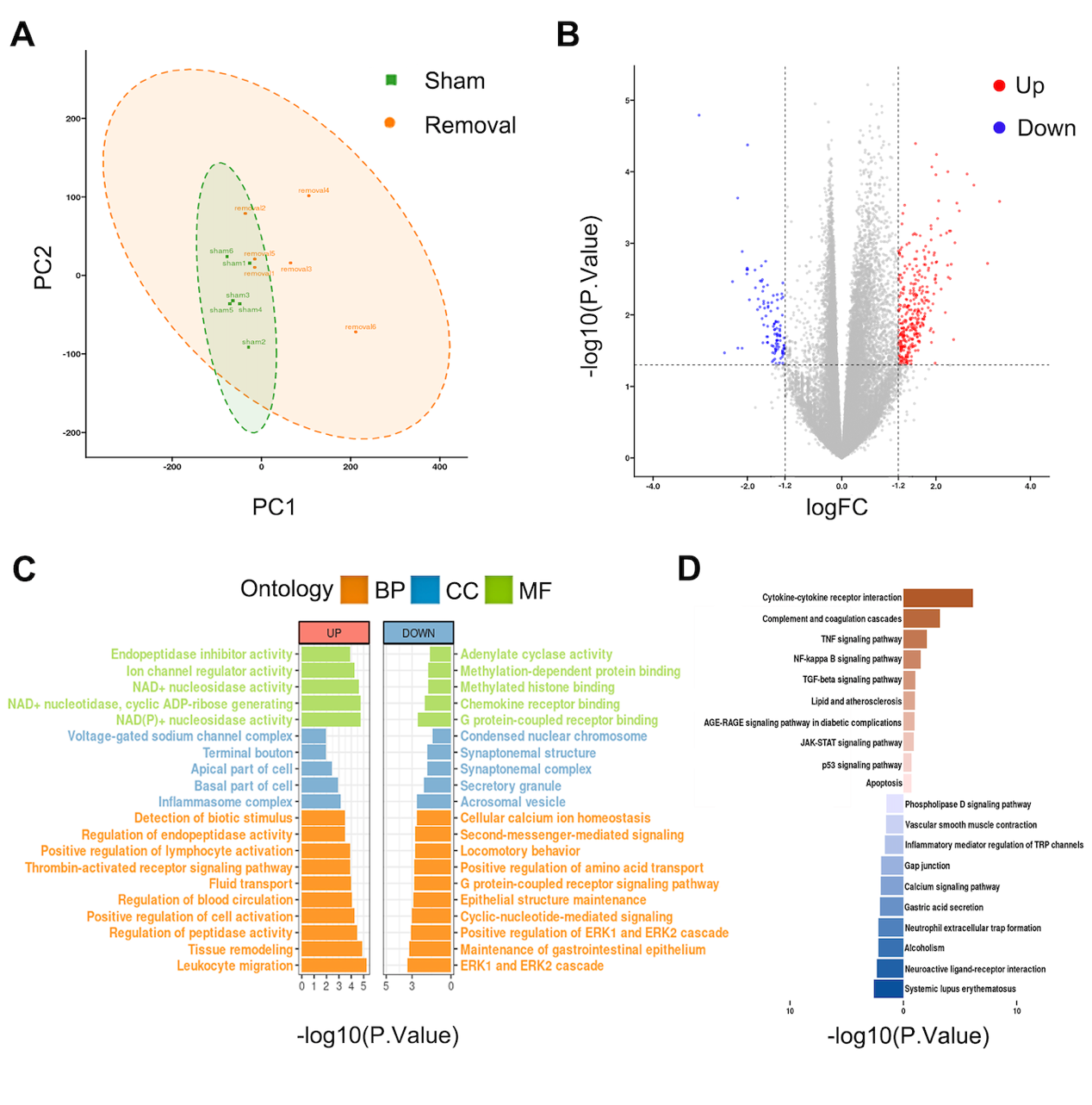


**S5 Fig. Unilateral lipectomy damages testis immune environment. a** PCA showing the distinction between the sham group and the removal group at the 4th week after unilateral lipectomy in 8-week-old male mice. PCA: principal component analysis, *n*=6. **b** Volcano plot showing the number of transcripts with upregulated and downregulated expression between the sham and removal groups in 8-week-old male mice. Log2 Fold Change ≥1.2. **c** Results of GO enrichment analysis of the genes with upregulated and downregulated expression between the sham and removal groups in 8-week-old male mice. *P.Value* < 0.05. **d** Results of KEGG enrichment analysis of the genes with upregulated and downregulated expression between the sham and removal groups in 8-week-old male mice. *P.Value* < 0.05.
